# Reforming the MRI system: the Israeli National Program to shorten waiting times and increase efficiency

**DOI:** 10.1186/s13584-021-00493-7

**Published:** 2021-10-18

**Authors:** Noga Boldor, Sharona Vaknin, Vicki Myers, Nina Hakak, Michel Somekh, Rachel Wilf-Miron, Osnat Luxenburg

**Affiliations:** 1 The Gertner Institute for Epidemiology and Health Policy Research, Sheba Medical Center, Tel Hashomer, Ramat-Gan, Israel; 2 Medical Technology, Health Information and Research Directorate, Ministry of Health, Jerusalem, Israel; 3 School of Public Health, Sackler Faculty of Medicine, Tel Aviv University, Tel-Aviv, Israel

**Keywords:** MRI, Health reform, Scanner, Waiting time, Israel

## Abstract

**Background:**

Long waiting times (WT) for Magnetic Resonance Imaging (MRI) are a challenge in many countries and demand is forecast to increase with ageing populations. Since MRI is essential for diagnosis in numerous medical conditions, timely performance is of the utmost importance.

**Objective:**

To describe the multi-faceted program developed by the Israel Ministry of Health (MoH) to shorten WT for MRI and increase efficiency, and to examine lessons that can be learned for other health systems.

**Data sources:**

Data were obtained from the Israel MoH from 2015–2019.

**Methods:**

The plan used multiple strategies and comprised the following elements: providing additional scanners, dedicating additional personnel to MRI units, maximizing efficiency, establishing a training program for radiographers and a fellowship program for radiologists, introducing financial incentives to health maintenance organizations and implementing a computerized monitoring system.

**Results:**

A substantial reduction in mean WT was demonstrated, from 52 days in 2015, to 24 days in 2016 and 2017. This was followed by a slight increase to 26 and 32 days in 2018 and 2019, respectively. The relative decline in WT from 2015 to 2019 was 38.5%. The number of scanners doubled during this period while the number of radiographers and radiologists with formal MRI training increased.

**Conclusions:**

The broad scope of this comprehensive reform was successful in addressing long WT and improving care provision from a wide perspective: economic, workforce and infrastructure. Bottlenecks in the MRI system cannot be addressed from a single angle, rather requiring a whole system approach.

## Background

Imaging tests are an important component of diagnostic processes, treatment planning and patient monitoring, and in many cases are an essential step in the diagnosis of conditions requiring immediate treatment. Among imaging modalities, MRI is a widely-used and highly advanced yet costly imaging test, due to expensive hardware and software, and expert manpower required to operate them. There is high demand for MRI due its unique innovative characteristics, the safety of its non-ionizing features (in contrast to computerized tomography (CT)) and its importance in diagnosis, including new clinical indications [1]. Furthermore, other factors such as the ageing population and rising incidence of chronic disease have also contributed to the growing demand for MRI in recent years throughout the world, including in Israel [2]. The substantial growth in demand for MRI, combined with a shortage of imaging personnel, created long WT, and a decrease in patient satisfaction in Israel. As a result, the Israeli Ministry of Health launched a national program aimed at improving accessibility and availability of MRI exams in the public healthcare system across the country, by implementing a multi-dimensional intervention, involving infrastructure, equipment, manpower and economic aspects.

In general, health policymakers are required to control health spending. Some Western countries adopted the ‘Choosing wisely’ approach which encourages both patients and clinicians to choose care that is necessary and to avoid superfluous tests [3]. In Israel, regulations were introduced in 1994 to create a balance between medical needs and economic constraints within which the health system operates. This methodology was based on the Certificate of Needs (CON) approach of cost containment, which puts licensing restrictions on the number of specialty medical devices, according to population size and healthcare needs. Under the CON, health care providers are required to obtain governmental approval prior to purchasing and operating new devices like MRIs, based on assessment of population needs. The logic behind this method, among others, is ‘induced demand’ [4], whereby increased supply leads to increased use, thus the CON may reduce expenditure and allow resources to be allocated where they are most needed. In countries which do not limit the number of machines, such as the US, Japan, Korea, Switzerland and Germany, there has been reported overuse of MRI exams [5].

Many countries struggle with long WT for MRI, which are often unevenly distributed across the country. To increase accessibility and availability of these tests, different countries implemented a range of strategies, adapted to the local healthcare system. For many years England faced long WT for diagnostic tests including MRI. This led the NHS to introduce a 6-week target for diagnostic tests [6]. The goal was achieved and WT has remained stable over the last decade. These changes were funded by a large increase in the NHS budget and included an extra 80,000 annual MRI scans commissioned through a mobile service, and an increase in the number of physicians and radiographers. From 2018 to 2019 the median WT for MRI varied from 19 to 25 days [7], and remained stable until February 2020, prior to the COVID-19 outbreak [8]. In 2018 the NHS commissioned the ‘Early Adopter’ imaging networks strategy with the aim of improving sustainability and service resilience, maintaining high quality training, retention of staff and flexibility [9].

On the other side of the continuum is Australia, dealing with possible underutilization of MRI devices. MRI in Australia operates under a licensing system and two factors are prerequisite for an MRI scan to be eligible for rebate: the scan type must be included on the Medicare Benefit Schedule (MBS); and the MRI machine must be fully or partially licensed [10]. The referral system allows general practitioners (GPs) to provide a referral only to partially licensed MRI machines for a limited range of tests. Only medical specialists can refer patients to a fully licensed MRI machine for the full range of MRI items, leading to a longer wait for specialist MRI referrals. This system of limiting Medicare rebates to particular machines greatly reduces patient access to MRI tests and consequently, might negatively affect population health. In response, an independent, clinician-led MBS Review Taskforce was established to ensure services are safe, high quality, up-to-date and better value for money for patients [11]. Increasing regional disparities also led to delayed diagnoses and treatment in certain regions. The average WT recorded in 2015–2016 was 8–10 days for GP referral, and 17–38 days for specialist referral [10]. In order to address long WT and regional disparities, the government expanded its MRI program in 2019 and added 50 new licensed scanners across the country, improving accessibility and availability [12].

Another country which dealt with long WT for MRI is Canada, with large variations between provinces. In 2018, the average WT for diagnostic MRI was 69 days, reflecting a limited number of scanners, below the OECD average. Many of the available scanners were older models, and the shortage of up-to-date equipment meant that patients faced longer-than-recommended WT. The overall impact of excess WT on the Canadian economy in 2017 was estimated at $1.4 billion [13]. WT recommendations were published for radiological services, based on priority levels and provincial capacity. In an effort to ensure imaging services reach those who need them most, the Ministry of Health decided to not only address the supply of equipment (which did not reduce WT) but also to focus attention on demand for MRI and possible over-use [14], in keeping with the Choosing wisely approach. The guidelines for MRI WT are ranked by severity: life threatening cases up to 24 h; urgent cases 7 days; partially urgent 30 days; and non-urgent 60 days. In the long term, demand for MRI services is expected to outpace growth in supply, resulting in a need for new scanners, with expected investment estimated at more than $3 billion over the next two decades [15].

In Israel, health services are provided within the National Health Insurance Law (NHIL). Israel provides high-level healthcare while successfully limiting costs [16]. Four health maintenance organizations (HMOs) are obligated to provide a uniform list of health services, known as the "Health Basket", for all members. The NHIL, enacted in 1995, established a system of direct oversight of the HMOs by the MoH, which itself administers certain services.

In 2015, the Israeli national average WT for MRI was approximately 52 days for the most frequent test (adult neurology MRI), and even longer for special tests, for example MRI in children requiring anesthesia [17]. International comparison in 2015 showed annual MRI tests rate of 34.7/1000 persons in Israel, compared to the OECD average of 64/1000. Furthermore, in 2015 the supply of MRI scanners in Israel (4.03/million) was substantially lower than the average of 15.8/million in OECD countries [18].

## Aim

To describe the multi-faceted program developed by the Israel Ministry of Health (MoH) to shorten WT for MRI and increase efficiency, and to examine lessons that can be learned for other health systems.

## Methods

At the end of 2015, the MoH decided to establish a National Program to shorten WT for ambulatory MRI exams. At the launch of the program, a WT target of 14 days for ambulatory MRI exam was publicly declared.

The process included identifying the barriers involved in long WT from three perspectives: economic, personnel and infrastructure. On the economic side, utilization of existing devices was examined, while calculating the cost of running second and third shifts of MRI examinations, in coordination with demand from the insurers (the HMOs). In terms of personnel, the shortage of skilled radiographers in the operation of MRI, the lack of both interpreting radiologists and funded radiologist positions in government hospitals, was mapped. In terms of infrastructure, the shortage of MRI devices and work processes resulting in sub-optimal utilization of the existing devices, were identified.

Several scenarios were calculated to estimate the number of devices required. The calculations took into account that the limiting factors in the provision of MRI exams were both an insufficient number of MRI scanners as well as the low availability of radiographers and radiologists with MRI-specific training. The personnel deficiency created a bottleneck in the system, which the mere addition of scanners could not have solved.

## Reform components

In order to meet these diverse challenges and strive towards achieving the 14-day target, the National MRI Reform was allocated a defined additional budget, including the following components:

- *MRI devices* The guiding principle was that every hospital with an emergency department and an existing CT device would have at least one MRI scanner. In 2015 there were 23 MRI scanners in Israel, and over a period of four years this number almost doubled, reaching 42 devices in 2019. Several additional licenses have since been issued with new devices expected to start operating soon.
- *Utilization of existing devices* MRI is an expensive imaging modality in terms of infrastructure and personnel required for its operation. In order to maximize the use of devices, working hours were updated where possible to 24 h active over 6 days for regular exams and to 24/7 for urgent testing. This meant that MRI scanners started operating around the clock, day and night, where necessary.
- *Personnel* Along with the addition of a large number of devices in a relatively short time, reinforcement of personnel—especially radiographers and radiologists, was required.
  *Radiographers* Each MRI device is operated by two trained radiographers. Prior to the reform radiographers gained on-the-job experience in MRI, however there was no formal training program. To address the shortage of skilled MRI radiographers, the MoH established The National Center for Imaging Professionals (NCIP). A 17-week training course combines theoretical studies, simulators and practical training. During the first four years of the project, 2016–2019, 110 radiographers received training at the center. Parallel to the training of personnel, additional positions for radiographers were allocated.
  *Radiologists* The first Israeli radiology fellowship program, organized and funded by the MoH in collaboration with the Scientific Council and the Radiological Society of the Israeli Medical Association, started in 2016. Prior to this, most physicians had to travel abroad to obtain a fellowship in radiology. The program addressed the shortage of specialist radiologists in various clinical fields and enables physicians to undergo a year’s local training in MRI. Over a period of half a year, more than 21 MRI units were recognized for fellowship in special fields of MRI (breast, muscle and skeletal imaging, neuroradiology, abdominal, cardiac, pediatric imaging). More radiologist posts were opened and funded by the MoH, to expand the capacity for MRI interpretation. From 2015 to 2019, 34 radiologists completed the fellowship program. In 2016 and 2018 fellowships were around half men and half women, while in 2017 and again in 2019, around 80% of those completing a radiology fellowship were women. The proportion of female radiographers undergoing MRI training also increased between 2016 and 2019 from 40 to 53%.
- *Computerized national database of MRI utilization* Until the end of 2015, MRI units reported activity via a structured form sent annually to the MoH including volume of exams performed and frequency of different clinical indications. A significant component of the reform was the establishment of a computerized system for reporting MRI utilization on a monthly basis across all medical facilities licensed to operate MRI. All data on MRI utilization is measured retrospectively, except for the first available appointment, which is measured with a prospective approach, and enables calculation of WT. The national MRI database provides comprehensive up-to-date data from all healthcare facilities in the country.
- *A financial incentive model* was introduced in order to increase the number of exams authorized and funded publicly by the HMOs and to shorten WT for MRI exams. A dedicated annual budget was allocated by the MoH for this purpose, divided between the four HMOs. The guiding principle of the incentive model was that the HMOs receive substantial economic incentives for increasing exam volume, which is expected to decrease WT until it reaches the median 14-day threshold. After two years of incentivized activity, a 30% increase in the overall number of publicly funded MRI exams in Israel was observed and WT were reduced to a mean 24 days. At that point, the economic model was reconsidered and its impact on the structure of the national "MRI market" reviewed. The new model, implemented in 2018, added incentives for the reduction of WT in addition to increasing exam volume. The economic incentives received by the HMOs are dependent on the accurate computerized documentation of MRI activity by the institutions that operate MRI scanners. The incentive model further included financial aid to hospitals without an MRI to purchase their first scanner.

For the calculation of WT, the most common MRI exam—adult neurology, which comprised the largest category or 36% of all exams in 2015, was chosen. The WT presented here was calculated—based on the stages of the reform implementation—in the following way:

- Pre-intervention: The average WT for 2015 was calculated through a telephone survey conducted in the second half of 2015. Secret shoppers asked for the first available date for an adult neurology MRI scan through the hospital appointment scheduling system. Average WT was calculated based on results from all MRI facilities.
- The average WT for the first available appointment for an adult neurology MRI exam from 2016 onwards is measured at the beginning of each month.

## Results

### Work processes and infrastructure

The main changes made during the first 4 years of the reform are summarized at two time points: 2015 (before the implementation of the reform) and after 4 years of implementation (Table 1).

**Table 1:** Changes made during the 4-year period of reform

	2015	2019
Number of MRI devices in Israel	23	42
Utilization of devices	Partial capacity	Gradual increase to full-time utilization—24 h over 6 days for regular exams and 24/7 for urgent testing
Number of radiographers trained in MRI operation at the NCIP	No formal national training program	110
Radiologists—positions	Hospitals were not incentivized for the addition of radiologists specializing in MRI	Funding of radiologist positions at all government hospitals
Recognition of units for MRI fellowship (as of 2016)	Few national MRI fellowships	21
Reporting	Annual hard copy forms including MRI utilization patterns	Monthly computerized, more detailed reports, including WT

### Waiting times

**Fig. 1:**
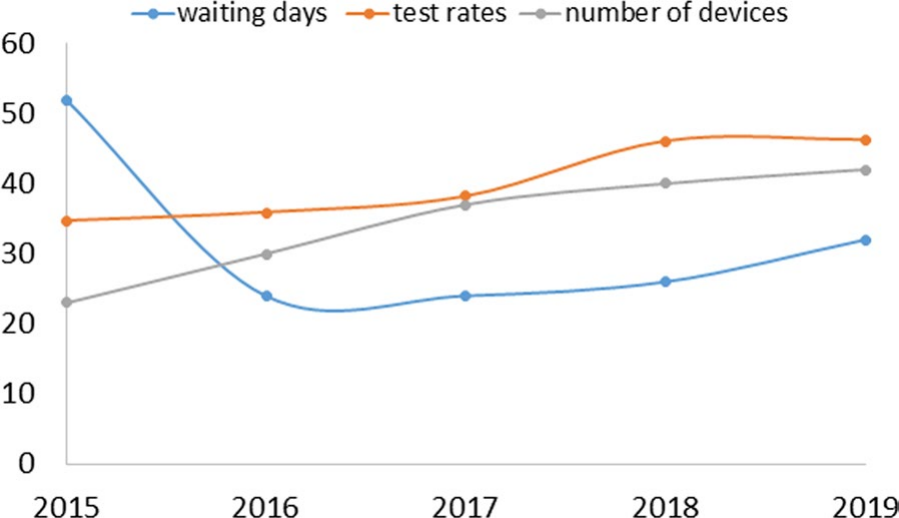
Average waiting time (days) for an adult neurology MRI exam, test rates (per 1000 people) and number of devices: Israel, 2015–2019

Since the beginning of the reform, the average WT for an adult neurology MRI decreased from 52 days in 2015 to 24 days in 2016, maintained in 2017. In 2018 and 2019 WT increased somewhat (26 days and 32 days, respectively (Fig. 1). From 2015 to 2019, WT demonstrated a 38.5% relative reduction.

### Volume of exams and patients

The per capita test rate increased from 35.3 tests per 1000 people in 2016 to 47.5 tests per 1000 in 2019: a relative increase of 34.6% (Fig. 1). In parallel the number of examinees (since some patients have multiple tests) increased from 32 per 1000 population in 2016 to 42 per 1000 in 2019, or a relative increase of 31%.

### MRI Devices

Since the start of the reform the absolute number of devices in the country almost doubled, from 23 to 42 devices (Fig. 1). Several additional licenses have since been issued with new devices expected to start operating soon.

### MRI devices per population

**Fig. 2:**
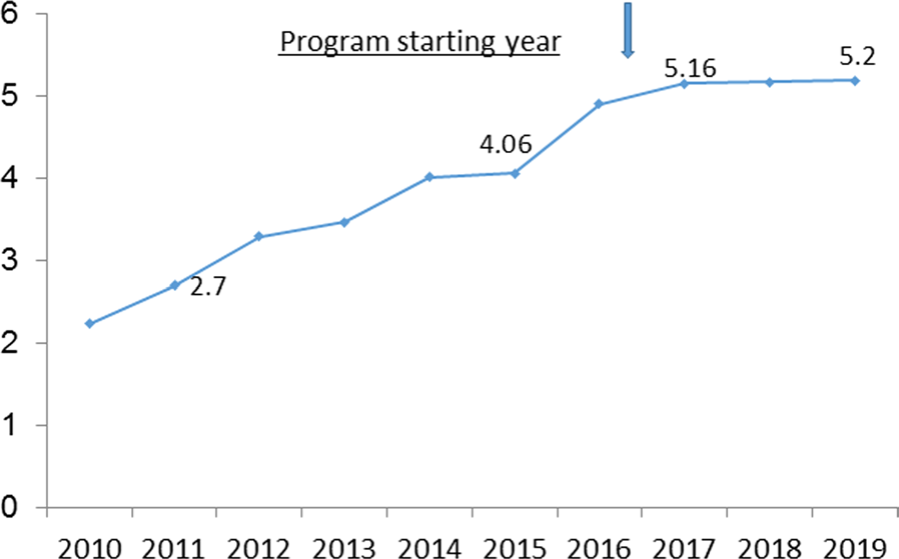
Rate of MRI devices in Israel 2010–2019 (number of devices per 1,000,000 population)

There was a steady increase in the ratio of devices per population that began before the reform and continued during the first years of reform implementation [18] (Fig. 2).

## Discussion

The national MRI reform was planned and launched with the main aim of shortening WT for MRI. The processes described in this article comprise a comprehensive multi-system MRI reform implemented for the first time by the Israeli MoH. The reform consisted of four pillars: (a) increasing the number of MRI devices; (b) creating economic incentives for the HMOs to increase exam volume, decrease WT, and help hospitals acquire scanners; (c) strengthening human resources by adding more positions, funding personnel, training radiographers and establishing a local MRI radiology fellowship and, (d) establishing a national database for ongoing monitoring of diverse process measures.

The combination of these tactics created a joint national mission, involving the MoH and health providers, aimed at improving accessibility and availability of MRI exams. The reform resulted in increased volume of examinations and decreased WT. The number of MRI scanners was doubled and utilization of scanners was improved to maximize efficiency. Manpower resources were amplified and training formalized, with positions added for radiology specialists and radiographers. Extensive training of personnel allowed the quality of exams to be maintained, and even improved, despite an increase in volume.

### Changes in WT

In the first two years of the reform there was a substantial drop in WT for MRI, and in the third year, once the market had stabilized, WT increased by 33% in the following two years. The subsequent increase in demand for MRI exams can be explained by the natural growth of the population and a continuous increase in the number of clinical indications as well a change in public expectations [19]. Similar trends were observed in other countries [14]. The target of 14 days—declared at the launch of the reform- has not yet been reached, however it is important to note that this goal is substantially shorter than those used in other countries [7, 12]. While average WT are still around a month, the system enables urgent tests to be performed within hours or a few days. Ongoing efforts are being made to further shorten WT, and current research is investigating whether the rise in tests is medically justified, as suggested by the Choosing wisely approach, and looking into the possibility of induced demand, that is increased utilization as a reaction to the increased supply of scanners and exams.

### Training and fellowship

Although accelerated training continues at the time of writing (October 2020), the Israeli healthcare system still experiences a shortage of both specialized radiographers and radiologists to perform and interpret MRI exams in a timely manner. This creates an imbalance between the availability of MRI infrastructure (scanners) and the growing demand for MRI exams. This imbalance is created due to different factors including the time required to train the increasing numbers of MRI staff and the difficulties in recruiting larger numbers of radiographers and radiology trainees.

A significant achievement of the program is that the majority of radiologists now have the opportunity to obtain a fellowship in Israel. This helps to keep senior physicians in the country instead of pursuing their training abroad, thus losing valuable medical personnel during the years of training. It is interesting to see that this fellowship program has encouraged more female physicians to undertake their training and become MRI specialists in Israel, comprising at least half of the fellowship trainees since the start of the program.

### Incentives

Striving to create balance in the health system, incentives were given to the HMOs which authorize imaging referrals for MRI. Based on the data collected in the system on exam volume and WT, economic incentives were adjusted annually. The incentives which the HMOs received (up to a certain threshold) facilitated a rise in the number of tests on the one hand, while controlling numbers on the other hand to mitigate an uncontrolled rise of exams en masse.

### IT infrastructure

The nationally coordinated computerized system established as part of the program, allows the monthly monitoring of a broad number of parameters, enabling more accurate surveillance and measurement of MRI activities at all sites and facilitating decision-making on an ongoing basis.

## Implications

Different countries have addressed the problem of long wait times for MRI with different tactics. According to the literature, Australia's strategy was to increase the number of scanners. The UK combined additional scanners and personnel to reduce WT. The strengths of the Israeli approach are that it tackled all aspects of the process, looking beyond the hardware and establishing a total system reform.

### What can be gained from the Israeli experience?

The Israeli MRI reform adopted a holistic, multi-faceted approach to address a wide scope of issues relating to accessibility and availability of MRI exams. Throughout its implementation, it has become clear that it is essential to continuously monitor its outputs, in order to address diverse challenges that require adaptation of the original intervention plan.

The MRI reform is a long-term ongoing process, continually updated, whose final fruits may not be seen for several years. Assessment of parameters at this point in the process indicates that the reform has achieved promising results and can be fine-tuned to succeed even further.

Leadership is crucial, especially in a publicly funded system. This reform was led at a national level by the MoH. As a regulator, the MoH has the ability to harness necessary government funds and achieve cooperation with professional bodies to establish professionally accepted regulations and licenses.

The combination of expanding the capacity of trained personnel and adding a large number of scanners, while increasing efficiency by lengthening operating hours, allowed an increase in the volume of exams performed and a parallel decrease in WT, the ultimate goal of the reform.

Economic incentives were integral to the program’s success, enabling the shortening of WT and the expansion of exam volumes over the four-year period.

The national MRI database provides comprehensive data from the majority of healthcare facilities, which are monitored monthly.

The plan for MRI reform is a long-term endeavor, which continues to evolve, while resting on stable foundations that guarantee the long-term maintenance of the reform.

### Limitations

Despite the notable achievements of the program there remain a number of challenges for the overstretched health system in Israel:

*Personnel for MRI performance and interpretation*: there is still a lack of trained personnel employed in the health system which needs to expand. Professional training requires investment of time and financial resources. This expansion of manpower should be implemented with a holistic view, beyond the specific needs of MRI.

*Device utilization:—*despite: increased efficiency in the use of scanners, some devices are still not used at their maximum capacity. Seven out of all facilities currently report capacity at 100% or more (based on at least 6 days per week, 20 h per day). According to OECD data Israel leads in the number of exams per scanner [20].

*Re-evaluation of the 14-day WT target*: Based on 4-years’ experience with the reform, different targets may be appropriate. Evaluation should be conducted every few years to determine public and expert opinion of acceptable WT.

*Measurement approach*: The system currently collects data for the first available appointment. This approach of calculating WT can cause bias, since it is sensitive to last minute cancellation and other unexpected events and might therefore, under-estimate WT. More accurate measurements, based on a larger number of available appointments, might be implemented [21].

## Conclusion

This comprehensive national plan led to improvements in the MRI system and reduced WT while remaining within the regulatory framework. The experience gained during the first years of implementation may be applicable to other countries, with a publicly funded national health system.

Bottlenecks in the MRI system cannot be adequately addressed from a single angle, rather requiring a whole system approach. A comprehensive, multi-system reform was able to improve service accessibility by combining economic, human resources and infrastructure perspectives. The program will continue to be monitored on an ongoing basis and adapted to the changing healthcare market, with longer term re-evaluation. We believe that to date the program has managed to concomitantly improve diverse aspects of service provision to create shorter waits for MRI, thus improving patient care, and will continue to do so.

With an ageing population and rising chronic disease, health systems must be prepared for continually increasing demand for MRI services. Future changes to the reform may consider taking into account patterns of demand for MRI. The COVID-19 pandemic is expected to further stretch health resources and have a significant impact on healthcare expenses.

## Data Availability

The data that support the findings of this study are available from the Ministry of Health but restrictions apply to the availability of these data, which were used under license for the current study, and so are not publicly available. Data are however available from the authors upon reasonable request and with permission of the Ministry of Health.
